# Supplementary material for: Toxicological Assessment of Oral Co-Exposure to Bisphenol A (BPA) and Bis(2-ethylhexyl) Phthalate (DEHP) in Juvenile Rats at Environmentally Relevant Dose Levels: Evaluation of the Synergic, Additive or Antagonistic Effects
Source: Int J Environ Res Public Health. 2021 Apr 26;18(9):4584. doi: 10.3390/ijerph18094584 (PMC8123661; doi:10.3390/ijerph18094584)
Supplement: Supplementary file 1 [file ijerph-18-04584-s001.zip › ijerph-1188519-supplementary.pdf]

Supplementary Table 1 - Primer sequences used in Real-time PCR analysis.

Gene		5' to 3' sequence
TSH	fw	GCCAGTGCAAAGTAAGCATGAA
	rev	CAGGCAGTAGGCACACTCTC
LH	fw	ACCTGGAATGCAAAAGCCAG
	rev	GTACTCGAACCATGCTAGGACA
FSH	fw	ATAGCCAACTGCACAGGACAT
	rev	TGGTGTAGCAGTAGCCCTCA
GAPDH	fw	AGTGCCAGCCTCGTCTCATA
	rev	GATGGTGATGGGTTTCCCGT
